# Intestinal Obstruction

**Published:** 1885-03

**Authors:** 


*Intestinal Obstruction.* By FREDERICK TREVES, F.R.C.S. Pp. 515. London: CASSELL & CO. 1884.

How is it that the taste of Messrs. Cassell, so exquisite in other walks, has led them into the dreadful primitive cærulean tint of the work now before us?

Our objection to the exterior of the book, however, does not apply to its interior; for it is undoubtedly one of the best of its series. Mr. Treves has set about his work in the true spirit of the scientist. He has formulated a definite arrangement of his facts at the beginning, and adhered to this arrangement throughout. He has collated the works of others, summed them up, tabulated and generalised them, and made his deductions from them with extreme patience and great ability. Nor are there wanting evidences of high-class original work. If we were to fix on one part of the work more excellent than another, we should select the chapters on Diagnosis, which, for abundance of detail and clearness of arrangement, we have nowhere seen surpassed.

At the present time, when the treatment of intestinal obstruction by abdominal section is pressing for solution, the appearance of Mr. Treves's work is most opportune. It is easy to foresee that the facts he records, and the by no means half-hearted advocacy which he puts forth, will have a powerful influence on the final settlement of this question.

